# Champion for Change Award: Promoting Rehabilitation in All Settings

**DOI:** 10.46292/1945-5763-29.suppl.xii

**Published:** 2023-11-17

**Authors:** B. Catharine Craven, Shane A. McCullum, Cesar Marquez-Chin, Kristin E. Musselman, Heather Dow

Dr. O'Connell is a most worthy recipient of the Champion of Change Award and joins an illustrious group of former winners with this distinction.

Dr. O'Connell is a Physiatrist, Professor in the Dalhousie Faculty of Medicine, and the Clinical Research Director, Institute of Biomedical Engineering at University of New Brunswick. Dr. O'Connell has demonstrated unwavering commitment in the field of physical medicine and rehabilitation. Dr. O'Connell is an inspiring leader who has been a global *catalyst for change*!

**Figure:**
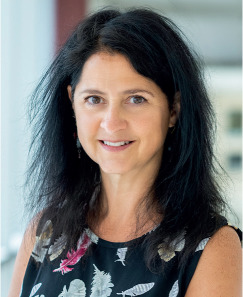
Champion for Change 2023 Awardee: Colleen O'Connell.

## Internationally

Dr. O'Connell is recognized internationally for her contagious laugh, her natural affinity to engage people and establish collaborations, and her determined advocacy for people living with spinal cord injury (SCI) in the community or disaster settings.

Dr. O'Connell has served on many global committees in the area of SCI during her career and is seen as a global expert in disaster rehabilitation. She has traveled to areas such as Haiti and Nepal within days following earthquakes to share her expertise and aid in disaster relief.

Furthermore, as the founder and Chair of the International Spinal Cord Society (ISCoS) Disaster Relief Subcommittee, she provided guidance and support for SCI management in disaster settings and most recently represented ISCoS in Ukraine.

In addition, Dr. O'Connell led the World Rehab Alliance Emergency Committee for the World Health Organization in 2022. She has represented Canadians at a number of international tables and brokered collaborations on behalf of other Canadians for over two decades. Further, she serves on the World Health Organization Steering Committee for the SCI Emergency Medical Teams standards. She has educated others on how to create temporary rehabilitation hospitals and triage and care for people with rehabilitation needs, and she has been instrumental in including rehabilitation services in post-disaster care teams globally. Dr O'Connell has also worked to prepare people with disability for emergencies by articulating the need for emergency preparedness and has had a strong voice in the SCI community during the COVID-19 pandemic and Canadian east coast hurricanes and flooding. She has represented the Canadian Association of Physical Medicine and Rehabilitation (CAPMR) and Canada at the International Society of Physical and Rehabilitation Medicine (ISPRM) since 2003. She is currently on the 2026 ISPRM Vancouver local scientific planning committee.

## Nationally

Dr. O'Connell's influence has not been confined to the global stage. On the national stage, Dr. O'Connell founded a nongovernmental organization that has been going for 20 years—Team Canada Healing Hands, which organizes and conducts international outreach projects with a focus on training in areas with identified needs for adults and children with disability providing education and rehabilitative care.

Dr. O'Connell served as the CAPMR President from 2007 to 2009. She received the CAPMR Award of Merit in 2015—the highest honor from our national specialty society. She subsequently served as the local organizer of the CAPMR annual meetings in New Brunswick, Newfoundland, and Prince Edward Island. Most recently, she has served as the Chair of Canadian Physiatry Research and Development Foundation for the last 3 years.

Dr. O'Connell is the local leader representing Stan Cassidy Centre for Rehabilitation (SCCR) in the National SCI Implementation and Evaluation Quality Care Consortium and is a former member of the Praxis Spinal Cord Institute Board of Directors.

Dr. O'Connell is the founder and lead of the Spinal Cord Injury Network of the Atlantic Provinces (SCINAPS), which was formed in 2018. The goal of SCINAPS is to bring together SCI stakeholders from the four Atlantic Provinces in Canada (New Brunswick, Nova Scotia, Prince Edward Island and Newfoundland and Labrador) to improve research and care from injury onset through transitions from acute care to rehabilitation to living in the community. The network includes approximately 80 stakeholders that meet three times per year to provide ongoing education to attendees and to plan for future collaborative projects. She has led and collaborated on several research studies intended to improve function and decrease the effects of common post-SCI secondary complications, many which involved acquiring and implementing emerging technology through research grants.

Dr. O'Connell has been a welcome presenter of “Top 6 Articles You Should Have Read” and workshop presenter throughout the history of the Canadian Spinal Cord Injury–Rehabilitation Association (CSCI-RA) and formally joined the executive of CSCI-RA in 2019.

## Locally

On the local stage, Dr. O'Connell is also a standout. She has been a Physiatrist and the Research Chief at the SCCR in Fredericton, New Brunswick, since 2000. In 2021, she was promoted to the role of Medical Director at SCCR while continuing to lead the Research Department and provide excellent care to her patients, and she is well liked by patients and families.

Dr. O'Connell is unique in her intuitive and ongoing preparation “for a pitch” and to actively listen and ask clarifying questions. For students, staff, and scientists in our audience, if you are not comfortable networking, we encourage you to watch Dr. O'Connell in action! She is engaging, knowledgeable, and likely to recruit you to assist in good work or supporting the local economy during a shopping venture!

**Dr. O'Connell's unique superpower is her ability to promote change through networking as she routinely advocates for a future where individuals of all ages and abilities, in all settings, feel empowered to participate in rehabilitation and pursue their passion! We are proud to have her as a driving force in the CSCI-RA.**

